# Erythrocyte sedimentation rate in canine leishmaniosis diagnosis: A new resource

**DOI:** 10.3389/fvets.2022.949372

**Published:** 2022-08-01

**Authors:** Maria Alfonsa Cavalera, Floriana Gernone, Annamaria Uva, Rossella Donghia, Grazia Carelli, Roberta Iatta, Andrea Zatelli

**Affiliations:** ^1^Department of Veterinary Medicine, University of Bari “Aldo Moro”, Valenzano, Italy; ^2^Unit of Research Methodology and Data Sciences for Population Health, “Salus in Apulia Study” National Institute of Gastroenterology “S. de Bellis” Research Hospital, Bari, Italy; ^3^Interdisciplinary Department of Medicine, University of Bari “Aldo Moro”, Bari, Italy

**Keywords:** acute-phase proteins, CRP, serum ferritin, ESR, dog, *Leishmania infantum*, active form, exposed

## Abstract

This study aims to evaluate the erythrocyte sedimentation rate (ESR) in *Leishmania infantum*-seropositive dogs compared with healthy dogs and to assess the existence of a correlation between ESR and clinical form of Canine leishmaniosis (CanL) as well as acute phase proteins (APPs). From October 2021 to January 2022, dogs were recruited in this study if *L. infantum*-seropositive by enzyme-linked immunoassay and classified as exposed or affected by a CanL active form based on physical examination, clinical score, and laboratory results [i.e., complete blood count, biochemical panel such as C-reactive protein (CRP) and serum ferritin, serum protein electrophoresis, and fibrinogen concentration measurement]. To evaluate the ESR of the dogs, a point-of-care device was used with a reference interval of 0–10 mm/h. Moreover, the ESR evaluation has been also performed in clinically healthy dogs, as control group. Thirty-six *L. infantum*-seropositive dogs [i.e., exposed (*n* = 10) and affected by CanL active form (*n* = 26)] were included in the study. Twenty-two healthy dogs were also enrolled. The mean value of ESR in dogs affected by a CanL active form was significantly higher than in exposed and healthy dogs (*p* < 0.0001). The ESR level was increased in 92% of dogs with CanL active form while positive APPs such as CRP, fibrinogen, and serum ferritin were increased only in 46, 48, and 58% of the animals, respectively. In exposed dogs, the ESR level was increased in 40% of cases. In dogs with active form, a significant positive correlation between ESR and total proteins, globulins, CRP, and fibrinogen, as well as a significant negative correlation between ESR and hematocrit, hemoglobin, and albumin/globulin ratio were detected. This study provides for the first-time data on ESR in *L. infantum*-seropositive dogs demonstrating dogs affected by a CanL active form have the highest ESR level and the majority of these dogs presented an increased ESR compared with exposed and healthy dogs. The evaluation of ESR by a point-of-care device proved to be a simple, inexpensive, and ready-to-use benchtop tool and ESR can be considered a helpful and timely inflammatory biomarker for the diagnosis of a CanL active form.

## Introduction

Canine leishmaniosis (CanL) is a sand fly-borne disease caused by *Leishmania infantum* and endemic in the Mediterranean basin (1). The systemic pathogenicity of this intracellular protozoan is strictly dependent on the complex canine host–parasite interaction (2). For example, a marked non-protective humoral immune response and an inadequate cellular immune response are associated with disease progression in susceptible dogs (3, 4). As a part of innate immunity, acute phase response (APR) has also been reported in CanL, being characterized by variations of acute-phase proteins (APPs) with increases in C-reactive protein (CRP), serum ferritin and haptoglobin, and decreases in albumin, paraoxonase 1 (PON1), or apolipoprotein 1 (Apo-A1) (5–10). Similarly, in humans with visceral leishmaniasis by *Leishmania donovani* and *L. infantum*, an inflammatory response is considered an ordinary feature, as indicated not only by the enhancement of APPs but also by the erythrocyte sedimentation rate (ESR) (11–13). This determination is a commonly performed laboratory test measuring the distance red blood cells travel in a tube of anticoagulated blood in a specific unit of time, most commonly an hour. In human medicine, ESR is usually used as a general index of illness and for tracking the occurrence and extent of inflammation. Though ESR is scarcely investigated in veterinary medicine, it has recently been found to be useful in cases of canine rheumatoid arthritis, osteoarthritis, babesiosis, and ehrlichiosis (14–17). For example, the potential value of ESR in combination with CRP as inflammatory biomarkers has been suggested for monitoring canine monocytic ehrlichiosis in dogs naturally infected by *Ehrlichia canis* (17). Therefore, this study aims to evaluate the ESR in *L. infantum*-seropositive dogs compared with healthy dogs and to assess the existence of a correlation between ESR and CanL clinical form as well as APPs.

## Methods

From October 2021 to January 2022, kennel and privately owned dogs of any sex, age, weight, and breed referred to the Medical Clinic Unit of the Department of Veterinary Medicine (Valenzano, Italy) were recruited in this study if *L. infantum*-seropositive by enzyme-linked immunoassay (ELISA). Dogs were excluded if suspected or known to be: (i) affected by diseases or treated with drugs able to influence the immune response and the inflammatory markers (e.g., neoplastic, auto-immune and heart diseases, diabetes mellitus and insipidus, hypo- and hyperadrenocorticism or hyper- and hypothyroidism, anti-inflammatory, and/or immunosuppressive drugs); (ii) affected by diseases able to influence the ESR according to the available scientific literature (i.e., canine rheumatoid arthritis, osteoarthritis, and babesiosis) (14–17). Furthermore, dogs were tested for anti-*Anaplasma phagocytophilum* (MegaCor Diagnostik, Horbranz, Austria) and anti-*E. canis* (Biopronix Agrolabo, Scarmagno, Italia) antibodies by indirect immunofluorescent antibody test and excluded in case of positivity.

Each dog underwent a complete physical examination, and a clinical sign-based score for CanL ranging from 0 to 19 was assigned (18). Blood samples were collected from either the cephalic or jugular veins and placed in K3 EDTA tube (2 ml) to undergo complete blood count (CBC) and ESR evaluation, as well as in sodium citrate (2.5 ml) to obtain plasma and perform fibrinogen concentration measurement. Moreover, a blood aliquot (5 ml) was placed in plain tubes to obtain serum after centrifugation (15 min at 1,500 × *g*) and perform biochemical panel including APPs (i.e., CRP and serum ferritin) and capillary zone electrophoresis. Dogs were tested for anti-*L. infantum* antibodies by ELISA (VetLine *Leishmania* ELISA kit, ref. LEIVT0310, Novatec, Dietzenbach, Germany). Based on physical examination, clinical score, and laboratory results, *L. infantum*-seropositive dogs were classified as “exposed” (i.e., absence of clinical signs and laboratory alterations compatible with CanL active form) or “affected” by a CanL active form (i.e., presence of clinical signs and/or laboratory alterations compatible with CanL) (3, 10). To evaluate the ESR of the dogs, a point-of-care device (MINIPET, DIESSE, Diagnostica Senese S.p.A., Siena, Italy) was used according to Militello et al. (19). The reference interval of ESR was established as 0–10 mm/h (19). Moreover, the ESR evaluation has been also performed in dogs considered clinically healthy (i.e., “healthy group”) based on physical examination, CBC, and biochemical panel, as control group.

### Data analysis

Dogs' characteristics are reported as mean ± standard deviation (M ± *SD*), and as frequencies and percentages (%) for categorical and their relative confidential intervals at 95% (*CI* 95%). Number and percentage of dogs suffering from a CanL active form according to combination of CRP and serum ferritin, and the ESR level have been evaluated. For testing the associations between independent groups (i.e., healthy, exposed, and active form of CanL), the Fisher's exact test for categorical variables was used, while the Kruskal–Wallis equality rank test was used to compare more than two independent groups. The Spearman rank correlation coefficient was used to test the strength and direction of association existing between two variables examined [i.e., between albumin, albumin/globulins ratio (A/G), CRP, ESR, ferritin, fibrinogen, globulins, hematocrit (HCT), red blood count (RBC), and total proteins]. Multiple comparisons were conducted for all possible pairwise on ANOVA or proportion, when necessary, with Bonferroni's adjustments.

When testing the null hypothesis of no association, the probability level of error at two tails, was 0.05. All the statistical computations were made using StataCorp. 2021. Stata Statistical Software: Release 17. College Station, TX: StataCorp LLC.

## Results

Thirty-six *L. infantum*-seropositive dogs (*n* = 19 males, *n* = 17 females) of different ages (7.6 ± 4.4 years) and breeds (24 were mixed-breed, 5 were English Setter, and one of each was Irish Setter, Épagneul Breton, Deutsch Kurzhaar, Akita Inu, Bull terrier, American Bully, and German shepherd) were included in the study. Based on clinical and laboratory evidence, these dogs were classified into two groups according to the CanL clinical form: exposed (*n* = 10) and active form (*n* = 26). Furthermore, 22 young (1 ± 0.5 years) and healthy dogs (*n* = 12 males, *n* = 10 females) of different breeds (*n* = 5 mixed-breed, *n* = 8 Pointer, *n* = 3 Bracco Italiano, *n* = 2 Central Asian Shepherd Dog, *n* = 1 Lagotto Romagnolo, *n* = 1 Pomeranian dog, *n* = 1 Maremmano-Abruzzese Sheepdog, and *n* = 1 English Setter) were also enrolled.

Mean, *SD*, and *CI* of ESR in the healthy dogs and the *L. infantum*-seropositive dogs were statistically compared and reported in Table 1. Mean, *SD*, and *CI* of the inflammatory markers evaluated (i.e., albumin, CRP, ferritin, and fibrinogen) and the globulins of the *L. infantum*-seropositive dogs were reported according to the clinical form in Table 2. Number and percentage of dogs suffering from a CanL active form according to the combination of CRP and serum ferritin values, and the ESR level are shown in Table 3.

**Table 1:** Mean (M), standard deviation (SD), and confidential interval (CI) of erythrocyte sedimentation rate (ESR) as well as number and percentage of dogs with altered ESR level in healthy dogs, exposed dogs, and animals affected by a canine leishmaniosis (CanL) active form.

Parameters^*^	**Status**				**Comparisons^ψ^**		
	**Healthy (*n =* 22) *(a)***	**Exposed (*n =* 10) *(b)***	**Active form (*n =* 26) *(c)***	***p*-Value^∧^**	***(b)* vs. *(a)***	***(c)* vs. *(a)***	***(c)* vs. *(b)***
ESR	9.09 ± 7.97 (5.56 to 12.62)^§^	9.50 ± 3.31 (7.13 to 11.87)^§^	23.11 ± 16.07 (16.62 to 29.61)^§^	0.0001^¥^	0.99	<0.001	0.01
ESR (%)	0.43 to 0.84^§^	0.30 to 0.90^§^	−0.02 to 0.18 ^§^	<0.001^∧^	0.99	<0.001	0.007
0–10 mm/h	14 (63.64)	6 (60.00)	2 (7.69)				
>10 mm/h	8 (36.36)	4 (40.00)	24 (92.31)				

* *Reported as: Mean and Standard Deviation (M ± SD) for continuous variables, and proportions for categorical*.

§ *Confidential Interval at 95%*.

¥ *Kruskal–Wallis test*.

∧ *Fisher's Test*.

ψ *p-value for comparisons on ANOVA, or p-value for pairwise comparisons of proportions, with Bonferroni adjustments*.

**Table 2:** Mean (M), standard deviation (SD), and confidential interval (CI) of positive and negative acute-phase proteins in exposed dogs and animals affected by a canine leishmaniosis active form.

	**Exposed** (***n =*** **10)**		**Active form** (***n =*** **26)**	
	**M ±SD or %**	**C.I. (95%)**	**M ±SD or %**	**C.I. (95%)**
Albumin	2.96 ± 0.27	2.84 to 3.08	2.56 ± 0.38	2.41 to 2.72
Albumin (%)		0.02 to 0.56		0.40 to 0.80
≥2.80 g/dl	8 (80.00)		10 (38.46)	
<2.80 g/dl	2 (20.00)		16 (61.54)	
Globulins	3.39 ± 0.25	3.21 to 3.57	4.53 ± 0.19	4.15 to 4.91
Globulins (%)		0.00 to 0.31		0.48 to 0.86
≤3.90 g/dl	10 (100.00)		8 (30.77)	
>3.90 g/dl	0 (0.00)		18 (69.23)	
CRP	0.08 ± 0.13	−0.01 to 0.18	0.83 ± 0.23	0.36 to 1.30
CRP (%)		0.00 to 0.31		0.26 to 0.67
≤0.45 mg/dl	10 (100.00)		14 (53.85)	
>0.45 mg/dl	0 (0.00)		12 (46.15)	
Ferritin	160.50 ± 42.10	130.38 to 190.62	378.65 ± 39.92	296.44 to 460.86
Ferritin (%)		0.00 to 0.31		0.37 to 0.77
≤270.0 ng/ml	10 (100.00)		11 (42.31)	
>270.0 ng/ml	0 (0.00)		15 (57.69)	
Fibrinogen	219.20 ± 115.48	136.59 to 301.81	346.44 ± 121.10	296.45 to 396.43
Fibrinogen (%)		0.002 to 0.44		0.31 to 0.72
≤335.0 mg/dl	9 (90.00)		12 (48.00)	
>335.0 mg/dl	1 (10.00)		13 (52.00)	

*As mean and standard deviation for continuous variable, percentage (%) for categorical*.

*C.I. (95%), Confidential Interval at 95%; CRP, C-Reactive Protein*.

**Table 3:** Number of dogs suffering from a canine leishmaniosis active form according to the combination of C-reactive protein (CRP) and serum ferritin values, and the erythrocyte sedimentation rate (ESR) level.

**Combo**	**Active form (%)**	**ESR**	
		**≤10 mm/h**	**>10 mm/h**
**CRP and ferritin**
CRP ≤0.45 mg/dl and ferritin >270 ng/ml	6 (23.08)	1 (50.00)	5 (20.83)
CRP >0.45 mg/dl and ferritin ≤270 ng/ml	3 (11.54)	0 (0.00)	3 (12.50)
CRP >0.45 mg/dl and ferritin >270 ng/ml	9 (34.62)	0 (0.00)	9 (37.50)

**Figure 1:**
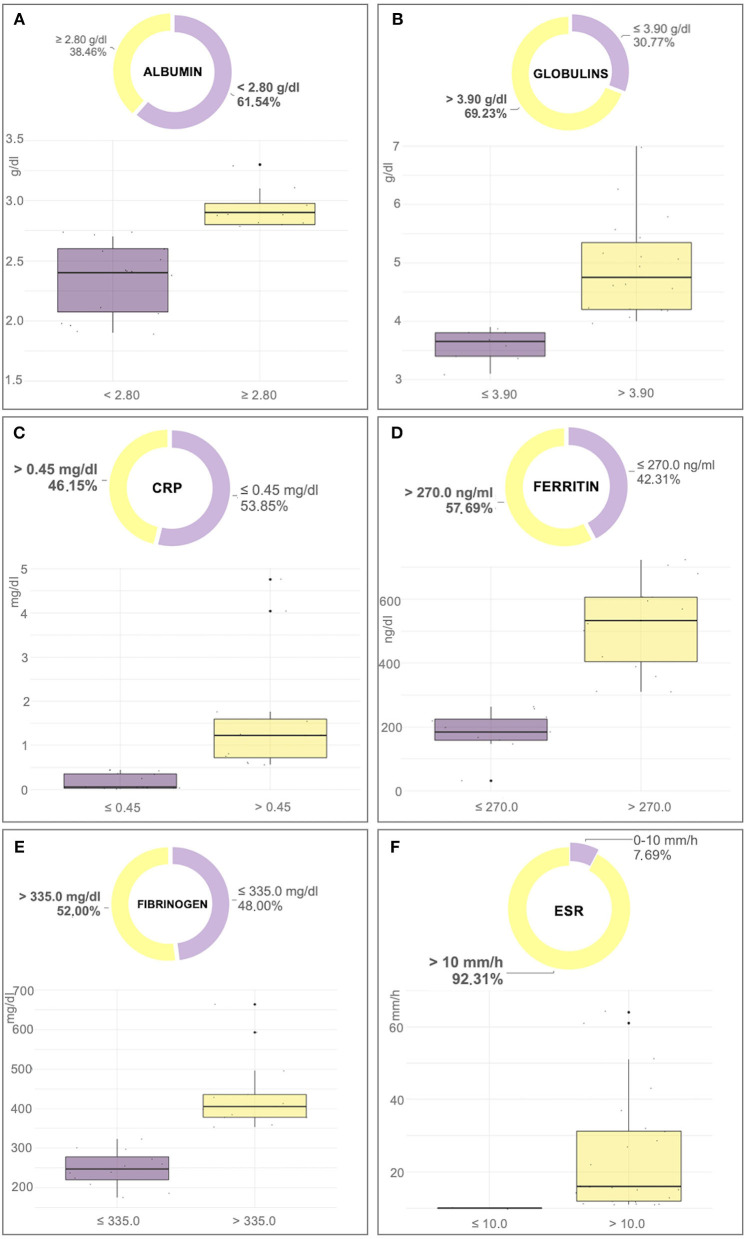
Box-plot and pie chart graphs of albumin **(A)**, globulins **(B)**, C-reactive protein (CRP) **(C)**, ferritin **(D)**, fibrinogen **(E)**, and erythrocyte sedimentation rate (ESR) **(F)** in dogs affected by an active form of canine leishmaniosis.

The mean value of ESR in dogs affected by a CanL active form was significantly higher than in the exposed dogs and in the healthy group (*p* < 0.0001; Table 1). Furthermore, the ESR level was increased in 92% of dogs with CanL active form while positive APPs such as CRP, fibrinogen, and serum ferritin were increased only in 46, 48, and 58% of the animals, respectively (Table 2; Figure 1). Reduced albumin and increased globulins were detected in 61 and 69% of dogs affected by CanL active form, respectively (Table 2; Figure 1). In exposed dogs, the APPs with altered levels were albumin and fibrinogen in 20 and 10% of animals, respectively, while the ESR level was increased in 40% of cases (Table 2).

Correlations between ESR and inflammatory markers in exposed dogs and dogs affected by active form are shown in Tables 4, 5, respectively. In exposed dogs, a significant positive correlation (*p* = 0.02) between ESR and fibrinogen was found (Table 4). In dogs with active form, a significant positive correlation between ESR and total proteins (*p* = 0.03), globulins (*p* = 0.01), CRP (*p* = 0.02), and fibrinogen (*p* = 0.001; Table 5), as well as a significant negative correlation between ESR and HCT (*p* = 0.05), hemoglobin (HGB; *p* = 0.02), and albumin/globulin ratio (*p* = 0.03; Table 5) were detected.

**Table 4:** Correlation matrix between erythrocyte sedimentation rate (ESR), hematocrit (HCT), hemoglobin (HGB), red blood cell count (RBC), albumin/globulin ratio (A/G), C-reactive protein (CRP), ferritin, and fibrinogen in exposed dogs (*n* = 10).

**ρ^¥^**	**ESR**	**HCT**	**HGB**	**RBC**	**Total proteins**	**Albumin**	**Globulins**	**A/G**	**CRP**	**Ferritin**	**Fibrinogen**
ESR	–										
HCT	0.16 (0.67)	–									
HGB	0.13 (0.71)	0.60 (0.06)	–								
RBC	0.18 (0.62)	0.09 (0.80)	**0.72 (0.02)**	–							
Total proteins	0.40 (0.25)	−0.39 (0.27)	−0.39 (0.26)	−0.39 (0.27)	–						
Albumin	−0.02 (0.94)	0.09 (0.80)	−0.04 (0.91)	−0.09 (0.80)	0.51 (0.13)	–					
Globulins	0.36 (0.30)	−0.53 (0.11)	−0.32 (0.37)	−0.29 (0.41)	0.58 (0.08)	−0.37 (0.29)	–				
A/G	−0.35 (0.32)	0.17 (0.64)	−0.07 (0.84)	−0.07 (0.84)	0.05 (0.88)	**0.81 (0.004)**	–**0.68 (0.03)**	–			
CRP	−0.21 (0.56)	0.50 (0.14)	0.17 (0.63)	−0.21 (0.55)	−0.58 (0.08)	−0.55 (0.10)	−0.09 (0.79)	−0.31 (0.38)	–		
Ferritin	0.31 (0.38)	0.51 (0.13)	0.42 (0.23)	0.08 (0.83)	−0.14 (0.69)	−0.30 (0.40)	0.14 (0.69)	−0.58 (0.08)	0.42 (0.22)	–	
Fibrinogen	**0.71 (0.02)**	0.58 (0.08)	0.31 (0.38)	0.15 (0.68)	0.05 (0.89)	0.07 (0.85)	−0.19 (0.59)	−0.13 (0.72)	0.00 (0.99)	0.45 (0.19)	–

¥ *ρ, Spearman's rho coefficient and relative p-value. The bold values indicate the statistically significant results*.

**Table 5:** Correlation matrix between ESR, HCT, HGB, RBC, A/G, CRP, ferritin, and fibrinogen in dogs affected by a CanL active form (*n* = 26).

**ρ^¥^**	**ESR**	**HCT**	**HGB**	**RBC**	**Total proteins**	**Albumine**	**Globulins**	**A/G**	**CRP**	**Ferritin**	**Fibrinogen**
ESR	–										
HCT	–**0.40 (0.05)**	–									
HGB	–**0.45 (0.02)**	**0.97 (<0.0001)**	–								
RBC	−0.30 (0.14)	**0.93 (<0.0001)**	**0.92 (<0.0001)**	–							
Total proteins	**0.43 (0.03)**	−0.16 (0.44)	−0.20 (0.34)	−0.21 (0.32)	–						
Albumin	−0.37 (0.07)	**0.43 (0.03)**	**0.44 (0.03)**	0.25 (0.22)	−0.16 (0.45)	–					
Globulins	**0.50 (0.01)**	−0.24 (0.24)	−0.30 (0.14)	−0.23 (0.27)	**0.88 (<0.0001)**	–**0.56 (0.003)**	–				
A/G	–**0.42 (0.03)**	0.33 (0.10)	**0.39 (0.05)**	0.26 (0.21)	–**0.57 (0.003)**	**0.81 (<0.0001)**	–**0.86 (<0.0001)**	–			
CRP	**0.48 (0.02)**	–**0.50 (0.01)**	–**0.57 (0.003)**	–**0.44 (0.03)**	0.23 (0.26)	−0.28 (0.18)	0.29 (0.16)	−0.36 (0.08)	–		
Ferritin	0.15 (0.46)	0.08 (0.72)	0.04 (0.85)	0.07 (0.72)	0.32 (0.12)	−0.23 (0.26)	**0.42 (0.03)**	−0.31 (0.13)	0.18 (0.39)	–	
Fibrinogen	**0.60 (0.001)**	–**0.57 (0.003)**	–**0.68 (0.0002)**	–**0.46 (0.02)**	0.37 (0.07)	**0.37 (0.0007)**	**0.60 (0.001)**	–**0.67 (0.0002)**	**0.42 (0.04)**	0.001 (0.99)	–

¥ *ρ, Spearman's rho coefficient and relative p-value. The bold values indicate the statistically significant results*.

## Discussion

This study provides for the first-time data on ESR in *L. infantum*-seropositive dogs compared with healthy animals and according to the CanL clinical form. Dogs affected by a CanL active form showed to have the highest ESR level and the majority of these dogs (92%) presented an increased ESR compared with exposed and healthy dogs. The increased ESR level detected in these animals is likely associated with an APR which is known to be characterized by a marked increase in APPs circulating in the blood, such as CRP and serum ferritin (5, 6). Red blood cell aggregation, one of the main determinants of ESR, is influenced by plasma proteins such as APPs whose increase reduces the negative electrostatic forces between red cells and facilitates erythrocyte aggregability, speeding up the sedimentation (20). Indeed, in this study, the ESR level was positively correlated to CRP, total proteins and globulins which are known to be increased in CanL active form (3), as well as to fibrinogen, being the most important driver for the development of large aggregates of red blood cells. Furthermore, an inverse correlation between ESR and HCT and HGB concentrations was found in dogs with CanL active form, confirming that if HCT is reduced, as in anemia which is a finding consistent with active disease, the velocity of upward-flowing plasma is altered so that erythrocyte aggregates fall faster (20).

Interestingly, though the positive and negative APPs were altered in dogs affected by a CanL active form as expected (e.g., CRP, fibrinogen, and serum ferritin were increased in 46, 52, and 58% of the animals, respectively), only the ESR level was increased in 92% of these dogs, potentially demonstrating a higher sensibility compared to the other inflammatory markers. Indeed, considering the combination of the two most used APPs for the classification and management of CanL according to the current scientific literature (i.e., CRP and serum ferritin) (21), only nine dogs suffering from a CanL active form presented both the analytes altered while ESR level was mainly always increased (Table 3). Therefore, the evaluation of ESR in dogs living in or coming from endemic areas for *L. infantum* with clinical signs and laboratory abnormalities compatible with active disease may represent a ready-to-use “watershed” for the veterinarian in deciding which diagnostic investigations to undertake.

In exposed animals, the ESR level was similar to that of the healthy group. Therefore, it could be assumed that in the exposed dogs' conditions leading to an increase in ESR are not present, as demonstrated by the absence in these dogs of inflammatory marker and red blood cell indices alterations.

A potential limitation of the present study can be represented by the small number of dogs enrolled due to the application of strict inclusion criteria. Furthermore, though ESR evaluation is demonstrated to be a simple, rapid, and low-cost diagnostic test, it represents a diagnostic tool at its infancy in veterinary medicine, requiring future studies to better define the physiological limits of this determination and the possible influences of factors such as age, gender, and reproductive status of dogs as already reported in human medicine. However, the significantly higher ESR level of dogs affected by a CanL active form compared with healthy and exposed dogs, herein described, would be unlikely altered by the revision of the normal range.

In conclusion, the evaluation of ESR by a point-of-care device proved to be a simple, inexpensive, and ready-to-use benchtop tool, and ESR can be considered a helpful and timely inflammatory biomarker for the diagnosis of an active form of CanL. Moreover, further investigations will be needed to assess if the identification and quantitation of the degree of the inflammatory response by the evaluation of the ESR might be also relevant for the therapeutic monitoring in CanL.

## Data availability statement

The raw data supporting the conclusions of this article will be made available by the authors, without undue reservation.

## Ethics statement

The animal study was reviewed and approved by Department of Veterinary Medicine of the University of Bari, Italy (Approval number, Prot. Uniba 24/2021). Written informed consent for participation was not obtained from the owners because the sampling performed in the study was part of the routine clinical visit planned for leishmaniotic dogs.

## Author contributions

AZ conceived, designed, and supervised the project. AZ, MC, FG, and AU performed the sampling. MC, AU, and GC performed the laboratory work. MC, RD, and AZ did the data analysis. MC wrote the first draft of the manuscript. MC, FG, AU, RD, GC, RI, and AZ revised the manuscript. All authors read and approved the final manuscript.

## Funding

This research and the APC were funded by DIESSE Diagnostica Senese Spa, Siena, Italy.

## Conflict of interest

The authors declare that the research was conducted in the absence of any commercial or financial relationships that could be construed as a potential conflict of interest.

## Publisher's note

All claims expressed in this article are solely those of the authors and do not necessarily represent those of their affiliated organizations, or those of the publisher, the editors and the reviewers. Any product that may be evaluated in this article, or claim that may be made by its manufacturer, is not guaranteed or endorsed by the publisher.
